# The influence of geometric nonconformance of the SB4 tension clamps on their strength and elasticity characteristics

**DOI:** 10.1038/s41598-024-80944-8

**Published:** 2024-11-28

**Authors:** Daniel Pieniak, Mirosław Guzik, Paweł Lonkwic, Piotr Lesiak, Jaroslaw Selech, Zbigniew Krzysiak, Jonas Matijosius, Marie Sejkorova, Artūras Kilikevičius

**Affiliations:** 1 Łukasiewicz Research Network-Institute for Sustainable Technologies, ul. Pułaskiego 6/10, Radom, 26-600 Poland; 2 Faculty of Transport and Informatics, WSEI University, 4 Projektowa Street, Lublin, 20-209 Poland; 3 The University College of Applied Sciences in Chelm, ul. Pocztowa 54, Chełm, 22-100 Poland; 4 Faculty of Civil and Transport Engineering, Poznan University of Technology, Institute of Machines and Motor Vehicles, Poznan, 60-965 Poland; 5 Faculty of Mechanical Engineering, Institute of Mechanical Science, Vilnius Gediminas Technical University, Plytinės Str. 25, Vilnius, 10105 Lithuania; 6 Department of Mechanical Engineering and Automation, Faculty of Production Engineering, University of Life Sciences in Lublin, Głęboka 28, Lublin, 20-612 Poland; 7 Department of Transport Means and Diagnostics, Faculty of Transport Engineering, University of Pardubice, Studentská 95, Pardubice, 532 10 Czech Republic

**Keywords:** Engineering, Materials science

## Abstract

The paper presents comparative laboratory investigations of tension fastener models that attach rails to concrete sleepers. The aim of the paper was to assess the influence of geometric nonconformity of the actual industrial product that meets the PKP Polskie Linie Kolejowe S.A. requirements on the operation of a tension-fastening clamp under stress. Due to the cost and limited possibility of research of the actual industrial product, an additional objective of the research was to validate the usefulness of it in the comparative assessment of the models. In the research, the authors used models of tension fastening clamps manufactured in incremental engineering technology (3D print) on scale 1:2. The properties of the fastening clamps (in their nominal shape) were compared (described in the PKP Polskie Linie Kolejowe S.A. documentation) with the fastening clamps of the actual shape. In the investigation, the authors have confirmed the negative influence of the non-conformance of the shape of the actual fastening clamps with the nominal ones.

## Introduction

The Railway transport is one of the modes that transports many types of cargo (hazardous materials included) and has a significant impact on the national economy. A positive impact of investments in railway transport on regional development was discussed in such publications as Brumercikova and Sperka^1^, Mankowski et al.^2,]^ and Dižo et al.^3^. Railway transport is also a part of the passenger carriage sector that performs regional and long-distance transits. Despite railway transport being one of the safer modes of transport, multiple research works have been published devoted to the safety^4–12^ and comfortability thereof^3,13,14^. When analysing the available literature, one can observe that safety in rail transport is heavily influenced by the transport infrastructure^4,15–21^, the appearance of different technical defects^22–25^. Various types of damage to the rail heavily influence the safety of the operation and lifespan of the rail infrastructure^26^. In terms of safety of the railway network, the most dangerous are the crossings of the railway tracks, with the roads (railway-crossing or level-crossing) being practically the only place of direct physical contact between otherwise relatively isolated transport modes^27,28^.

Adopting a certain fastening clamp stiffness and damping can impact the rail frequencies, vibration amplitude, wave propagation velocities and attenuation. Optimising the fastening clamp parameters can potentially reduce short-pitch corrugation and wheel roll noise in the relevant frequency range^29^. The non-linear behaviour of the rail fastening system can cause large displacements that exceed the allowable limits, even if the sleeper track is designed to meet the current serviceability evaluation methods at the ends of the railway bridge^30^. The fatigue life of the fastening clamp is affected by its assembled state and the excitation frequency. The resonance at the second natural frequency decreases the fatigue life of the fastening clamp, even though its vibration fatigue strength is generally sufficient. The fatigue damage peril points shift from the inner heel towards the lateral leg as the excitation frequency increases, regardless of the assembled state and the excitation amplitude^31^.

The appearance of bolt failure during inspection can be attributed to a fatigue fracture induced by the stress concentration resulting from a model selection error on the full thread bolt. The absence of a steel pad resulted in an increase in vertical dynamic stress caused by the trolley through the rail gap. The Finite Element Method (FEM) analysis of the trolley rail system uncovered a behaviour like a seesaw, which led to an elevation in the eccentric tensile stress experienced by the bolt’s pad-support area. Stress levels exceeded the material’s yield strength, specifically in the bolt adjacent to the gap in the rail pad. This observation aligns with the findings of the finite element method (FEM) and the location of failure^32^.

The AHP method, despite its complexity, is accurate in multi-criteria decision making. Aided by software tools, it reduces subjectivity and produces relevant data. The method was applied at the Štúrovo railway station, and can be used for any station in the ŽSR network. Cluster analysis was used in the BDŽ network for passenger transport and could be a useful tool for future research^1^. Statistical data was used to determine the probability of failure-free operation for three types of rail fastenings - ZBR-Sh, ZBR-PShM and ARS. Each type had specific failures identified, with the ZBR-PShM being the most reliable and the ARS the least reliable. Taking into account factors such as reliability, durability, and maintenance costs, the ideal area of deployment is suggested based on the line plan and cargo intensity^33^.

A model was developed to simulate the coupling between high-speed rail vehicles and tracks, focussing on the effects of random track irregularities and the WJ-8 fastening system. Dynamic responses and the stress time history of the fastening clamps are calculated using the model and fatigue damage analysis is carried out using the rain-flow counting method and cumulative damage theory to evaluate the impact of track irregularities on the probability of fatigue failure of the fastening clamp over time^34^. Despite a significant decrease in the response amplitude of a high-damping composite fastening, it improves the dynamic fatigue life of the fastening clamp and allows for cost savings in the fastener system^35^. The finite element method was applied to analyse the effects of the loading frequency, preload and stiffness of the fastening components on the damping and stiffness of the Rail Web Fastening System and Rail Foot Fastening System. All components of the fastening systems were considered in the models^36,37^.

**Fig. 1:**
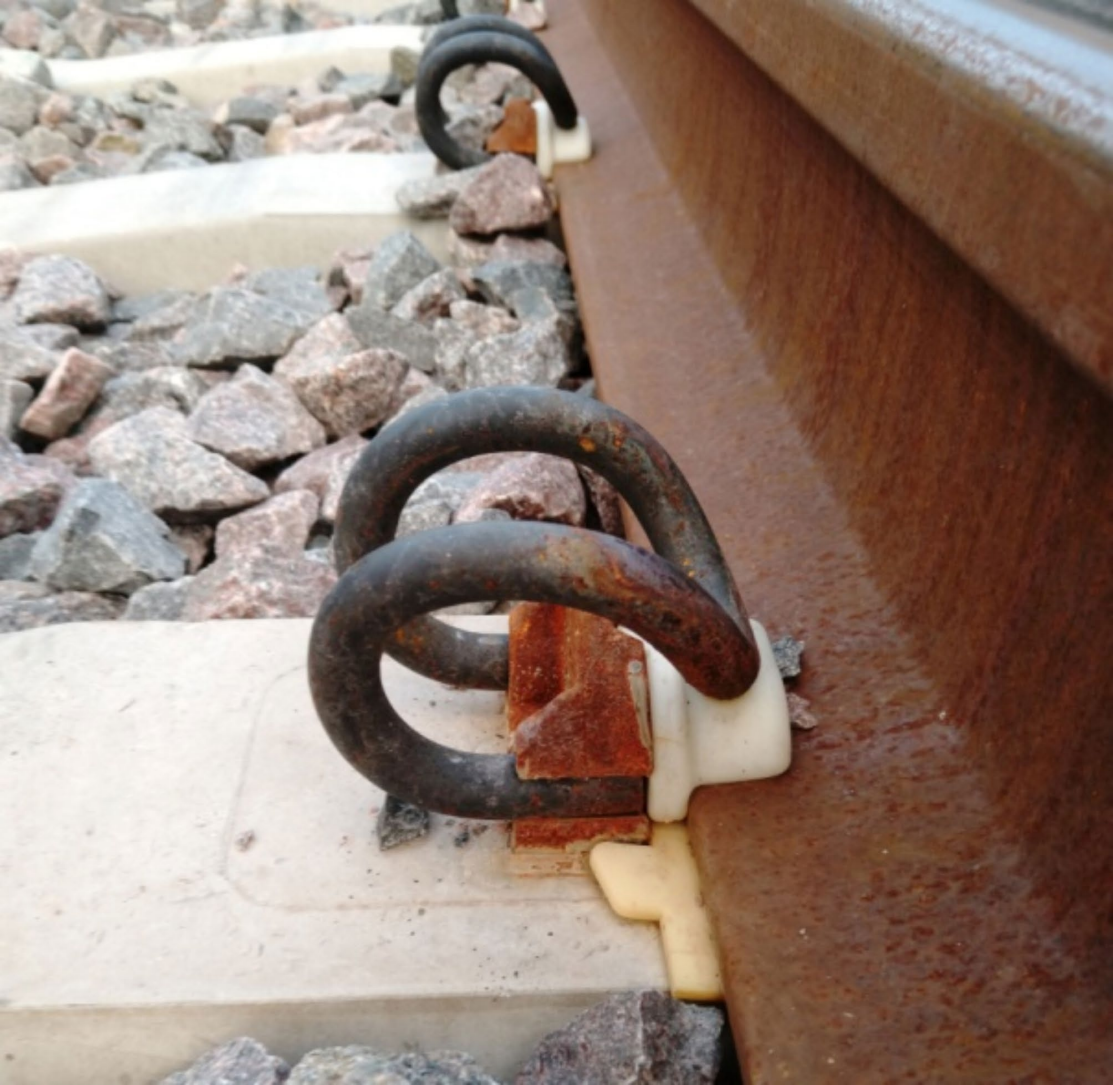
An image of the SB type fastener.

As mentioned above, the transport infrastructure is most vital to the safety of railway transport; therefore, a good technical condition of the railway track is particularly important in this respect. The fundamental elements are the rail, the sleepers, the fasteners, and the track. The attachment of the rail to the sleepers is necessary to maintain the integrity of the construction and carry the load generated on the railway track. This task can be accomplished with the use of tension fasteners. The most important component of the fastening system is the tension-fastening clamp, which can be classified as a resilient element. Tension fasteners of different types are objects of investigation, the results of which are closely related to the geometry of the investigated elements. Such investigations include laboratory mechanical tests^38,39^, including fatigue tests^40^ and FEM clamp tension modelling^41^. SB tension clamps are components of rails used by PKP Polskie Linie Kolejowe S.A. (PKP PLK S.A). The SB tension fasteners (Fig. 1) superseded the complex K type fasteners. Each of the K-type fasteners was composed of 34 elements for each sleeper, and the weight of the entire set of elements reached approximately 20 kg^42^. The clamps are elements of the fastening system and carry static and dynamic impulse stresses. In Vossloh^43^ the authors confirmed that SB4 are characterised by high elasticity and the potential for use on high-speed/load rail tracks.

SB clamps are made of hot drawn and rolled 50S2 steel bars with a diameter of 16 mm. The nominal dimensions of an SB4 clamp are precisely determined in the PKP PLK S.A. document titled ’Technical conditions of production and acceptance of tension clamps and springs that attach the rail to the sleeper/switch sleeper’ (NR WTWiO - ILK3D-5183-5/2007E.P)^44^. In this document, in addition to the dimensions of the steel bar, parameters such as the radiuses of the bending arms, the the nonparallelism of the arms and the diameter of the the flexure of the diameter of the bar at the clamp were determined. The SB4 clamps represent the latest, yet little known, design used by Polish railways. They account for changes in geometry and their mounting in the track compared to the older SB3 model. These changes are the result of many years of operational experience on railway lines of various categories, starting with the main lines. During this time, breaks to the clamp arm and permanent operational deformations were observed. The deformations were also the result of repeated disassembly and reassembly necessary when replacing damaged rails.

The Latvian railway uses KB and Vossloh rail fastenings. The KB fastenings have been popular for more than a decade and require separate bolts to connect the rail sole and the lining to the cross-link. The Vossloh fastenings are newer and use a spring to hold the rail sole and the crosstie together with a single bolt. A debate over which fastening is better has continued for years^45^. The 60Si2Mn steel bolt fractured during its 8-year lifespan, despite having a satisfactory microstructure, hardness level, and depth of the decarburization layer. Inclusions were not found to be the cause of the fracture^29^. To ensure accurate results, the specimens were subjected to the same thermal and shotpeening treatments as the actual fastenings in the same industrial facility. The study used a probabilistic approach to analyse the fatigue performance of the material known as the P-S-N approach^31^.

The SB4 tension clamps made by different manufacturers were visually compared. It turned out that in some cases, the products did not conform to the PKP PLK guidelines. It was assumed that the non-conformance of the actual dimensions and shapes of the SB4 clamps with the nominal ones, as specified by PLK, may have an impact on their durability and operation. The research problem undertaken appears to be notable because, as a matter of principle, elastic elements are applied to accumulate and dissipate energy, regulate stresses, damp vibration, and attenuate excess stress^46,47^. The clamps also play a role in the mutually controlled displacement of the connected elements^46^. Therefore, their design and operation are crucial in light of their role in the entire fastening system.

In light of the above, the aim of the investigation was to evaluate the influence of geometric non-conformance of actual industrial products with nominal requirements on the operation of the tension clamp under stress.

## Methods

**Fig. 2:**
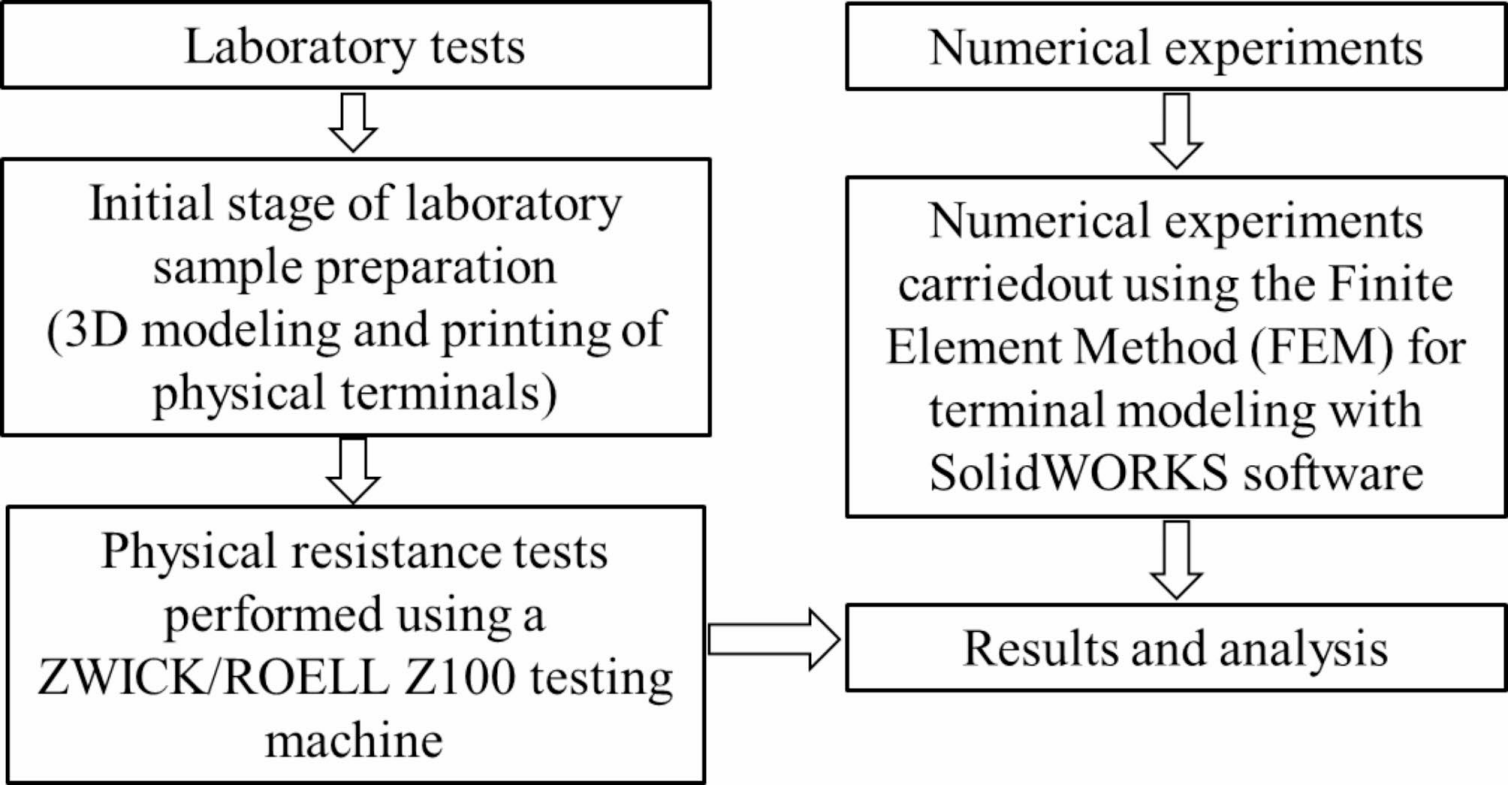
Flowchart of laboratory and numerical experiments.

Figure 2 delineates the primary phases of the studies, beginning with the first 3D simulations and ending with the results. These supplementary visual data enhance the comprehension of the study process and methods, particularly the distinctions and interconnections between laboratory testing and numerical experiments.

The material for the numerical model was determined individually based on the available data for the 50S2 material, which is the same as that used for the actual clamps. The boundary conditions of the numerical model were reflected on the basis of the actual usage conditions. The method of fixing the clamp in the numerical model, as shown in Fig. 1, was represented as cylindrical surfaces depicted in Fig. 7b. On this surface, all degrees of freedom were restricted by fixing these surfaces in place.

Due to its elasticity, the clamp applies a point load on the guide surface (Fig. 1). Due to the challenge of modelling contact stresses in SolidWORKS 2023 software, this method of clamp loading was represented in the numerical FEM model as a load applied to a small surface located at the top of the clamp’s curvature (Fig. 7b).

**Fig. 3:**
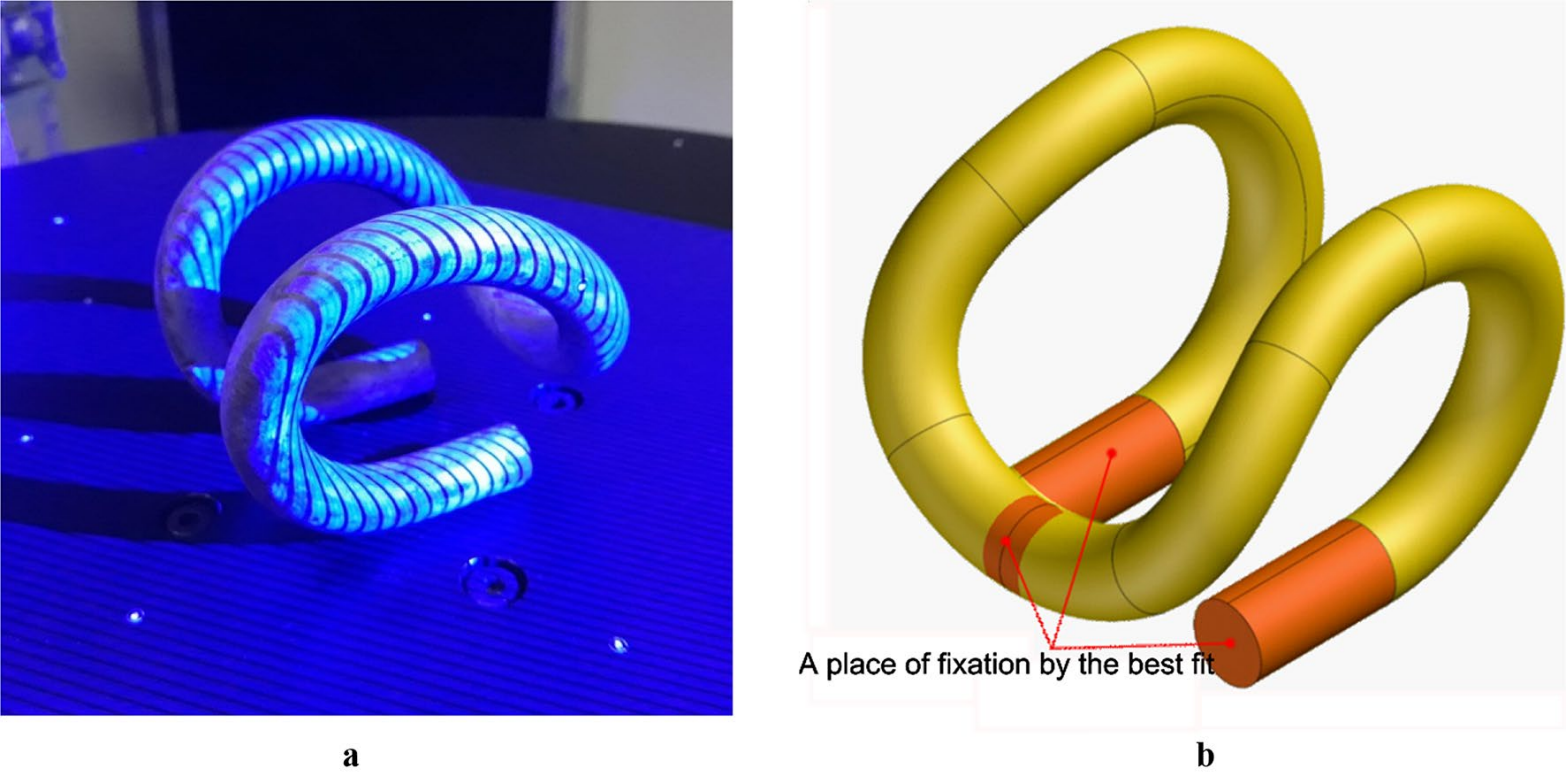
(**a**) SB4 tension clamp during scanning (**b**) The homing method.

Geometric measurements of tension clamps were made in a sample group of 4. Direct measurement methods are problematic if the objects are made following 3D bending of a thick wire and do not provide full information regarding their shape. Therefore, for the measurements, the authors used a 3D GOM Atos III triple optical scanner (Fig. 3a). In the GOM Inspect software, the authors compared the geometries of the 3D models of the actual clamps obtained during the scanning with the CAD reference model reproduced in Solid Edge based on technical documentation^44^.

In PKP PLK S.A. guidelines^44^ regarding direct measurements, no main measuring bases were determined. Therefore, for analysis, the authors adopted such a matching method of comparable models that best fits the direct measurements of the most fundamental toleranced dimensions. A best-fit fixture was applied of the area of the clamp straight ends (Fig. 3b) mating with the fastening anchor as well as fitting of the frontal cross section of the arch mating with the rail foot.

**Fig. 4:**
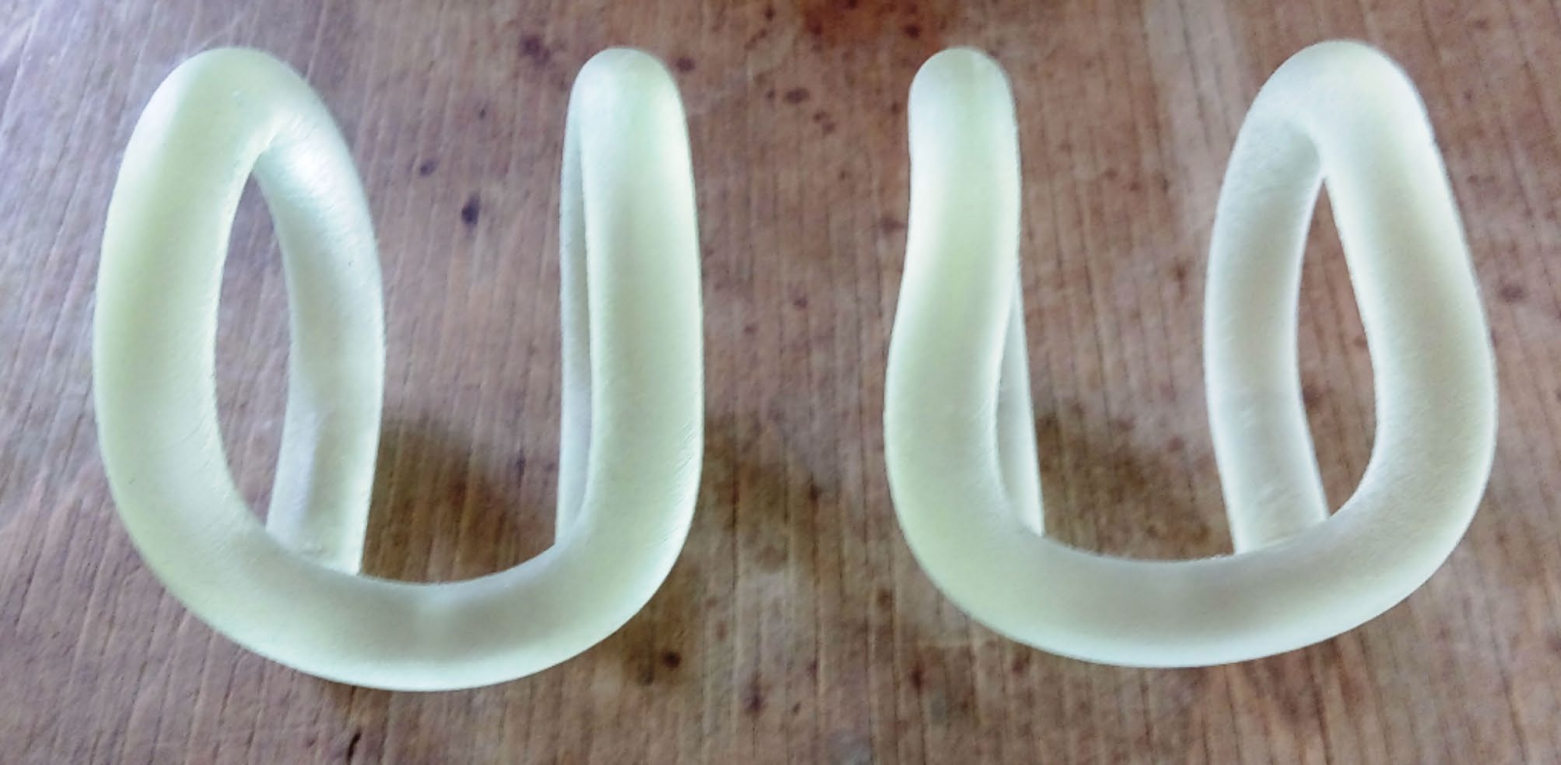
Models of SB4 tension clamps made in 3D technology (from left to right: the model conforming with the nominal and the model of the actual product).

Then, using an OBJET EDEN 500 V rapid prototyping system and RGD-720 photopolymer resin, two series of samples were printed: one corresponding to the solid CAD model of the nominal tension clamp and the other corresponding to the triangle mesh of a selected real clamp. To reduce the cost while preserving the optimum count of the samples, the physical models of the clamps for destructive strength tests were reduced twice in size when 3D printed (Fig. 4). Due to the lack of real clamps that would fully represent the nominal shape specified in the PLK documentation, comparative investigations were carried out on substitute physical models.

The investigations of the mechanical properties of the SB4 clamps were performed on a ZWICK/ROELL Z100 material testing machine. In the tests, the authors used a nominal rating load cell of 10 [kN] and accuracy classification 0.5 (in accordance with ISO 7500). Deflection measurements were carried out with the use of the makroXtens macroextensometer according to ISO 9513^48^. The Test Expert II programme was used to develop test load courses. In which the load was processed in cycles. In the first stage, the authors determined the strength and elasticity characteristics of the model material applied to reproduce the clamps. Strength tests of the RGD-720 resin were carried out according to the ISO 527-1:2012 technical standard^49^. The tensile strength was determined to be the highest value of tensile stress. The calculations were made on the basis of the following equation:

1 $$\sigma _{M} = \frac{{F_{m} }}{{A_{0} }}$$

where:

$\:{\sigma\:}_{M}$ – tensile strength [MPa],

$$\:{F}_{m}$$ – maximum force [N],

$\:{A}_{0}$ – initial surface area of the sample [mm^2^].

Furthermore, the authors determined the strain ε_M_ corresponding to the maximum force and stress ($\:{\sigma}_{B}$) and strain ($\:{ \varepsilon }_{B}$) at the moment of fracture. Elastic moduli were determined in the strain range from 0.05% with the tangent method. The calculations were made according to the following equation:

2 $$E_{T} = tg\alpha _{T}$$

where:

$\:{E}_{T}$ – longitudinal modulus of elasticity (Young’s modulus) [MPa],

$\:tg{\alpha}_{T}$ –slope of the tangent line to the stress-strain curve.

The tensile work was derived from the following equation:

3 $$\:{W}_{M}=\underset{{l}_{0}}{\overset{l}{\int}}F(l-{l}_{0})$$

where:

$\:{W}_{M}$ –tensile work to maximum force [Nmm],

$\:F$ – stress during the test [N],

$\:{l}_{0}$ – initial length of the sample [mm],

(*l* - *l*_0_) – elongation of the sample [mm].

**Fig. 5:**
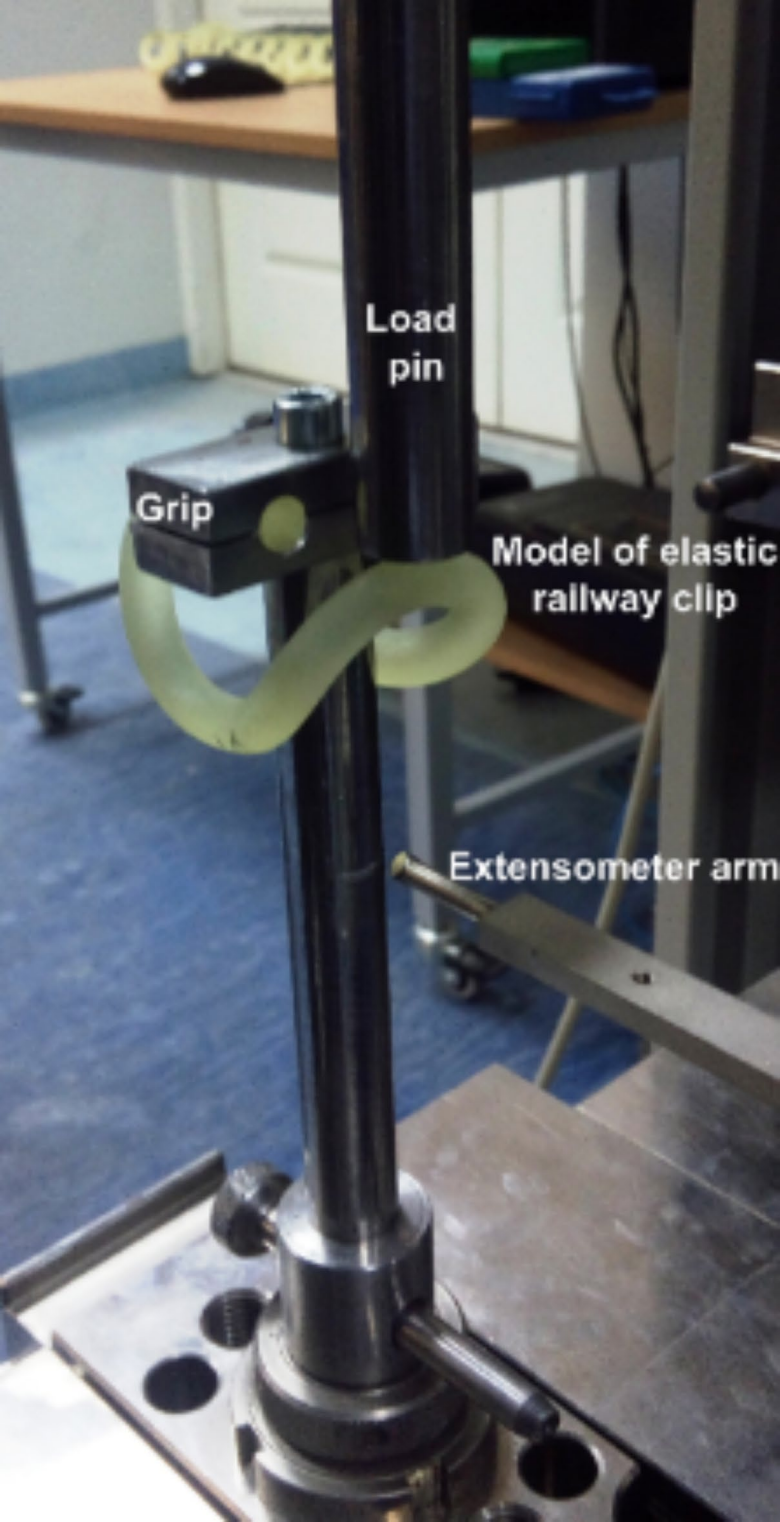
SB4 clamp testing configuration.

From the stress-strain curve, the authors determined the work performed until the fracture $\:{W}_{B}$. In the next stage, the authors carried out comparative tests of the mechanical properties of the models of the 3D printed clamps (Fig. 4). The strength and elasticity of the clamps were determined on the ZWICK/ROELL Z100 testing machine. Special equipment was applied (Fig. 5) designed and manufactured specifically for the research discussed, according to the guidelines contained in the PKP PLK documentation^44^. The equipment allowed us to apply a vertical stress to the clamp. The bending of the clamp was recorded with the makroXtens mecrotensometer (Fig. 5).

The clamps were tested with two methods, varying the stress characteristics. Monotonic stress was applied to the clamps, allowing their elastic characteristics to be determined. The tests were continued until fracture to determine the final strength of the clamp. Then, the authors continued the tests aiming at determining the work characteristics under nonmonotonic elastic stress. The course of the non-monotonic elastic test stress was to reflect the real world stress conditions. The test covered the initial stress until the clamp bending (4 mm), which corresponded to the initial fitting stress. The stress bending the clamp by 4 mm was applied for 120 s and then an impulse dynamic vertical stress was applied with a velocity of 200 mm/min that bent the clamps to 6 mm and, without interruption, the stress was reduced to the bending of 4 mm and maintained for 120 s. Then, the stress on the sample was released. Furthermore, the authors determined the stress-strain loop under monotonic stress conditions that corresponded to the strain of 4 –6 mm – 4 mm. Stress work was recorded for these types of stress. In general, the test parameters were selected in accordance with the guidelines contained in the PKP PLK^44^ document but were extended and modified to suit the scale and material of the model clamps.

### Geometric analysis

Comparison of the nominal CAD model of the SB4 tension clamp with the 3D scans of the actual objects discloses a significant simplification of the arches in the frontal area. Based on the measurements of the scans of four clamps, in the dimensional quality control software, the authors observed that the average deviation of the R20 dimensions is approx. +14 mm, and the R35 dimensions are approx. 5 mm (Table 1).

**Table 1:** Deviations (mm) of the measured radiuses of the arches in the frontal area^50^.

Clamp number	Arch R35, tol. ±2 mm	Arch R20L, tol. ±2 mm	Arch R20P, tol. ±2 mm
1	-5.04	+ 15.66	-3.02
2	-5.10	+ 13.38	+ 13.30
3	-5.09	+ 6.78	+ 14.38
4	-5.04	+ 14.51	+ 32.77

**Fig. 6:**
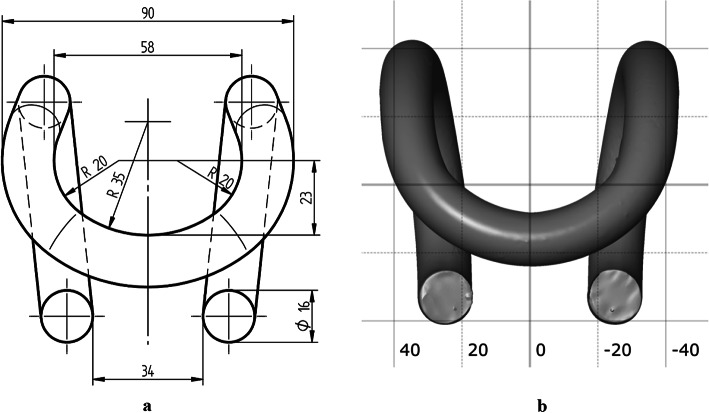
(**a**) Nominal dimensions of the CAD model of the SB4 clamp- frontal view (**b**) Triangle mesh of the actual SB4 clamp – frontal view.

According to the documentation these dimensions are toleranced in the range ± 2 mm, but still, they are not subject to partial examination of shape, dimensions, and workmanship tolerances specified in the said WTWiO documentation^44^. Figure 6a presents a technical drawing based on the CAD model reproduced from the technical documentation. Figure 6b presents the view of the triangle mesh of the actual and nominal SB4 clamp. A different shape of the clamp in the frontal area can already be seen when visual comparing the two drawings.

We can clearly observe the enlarged radius of the lateral arches, whose nominal value is 20 mm, and a decrease in the central radius of the arch, whose nominal value is 35 mm. No excess deviations were observed in the dimensions of the arches that define the clamp profile^50^.

### SB4 tension clamps FEM analysis

**Fig. 7:**
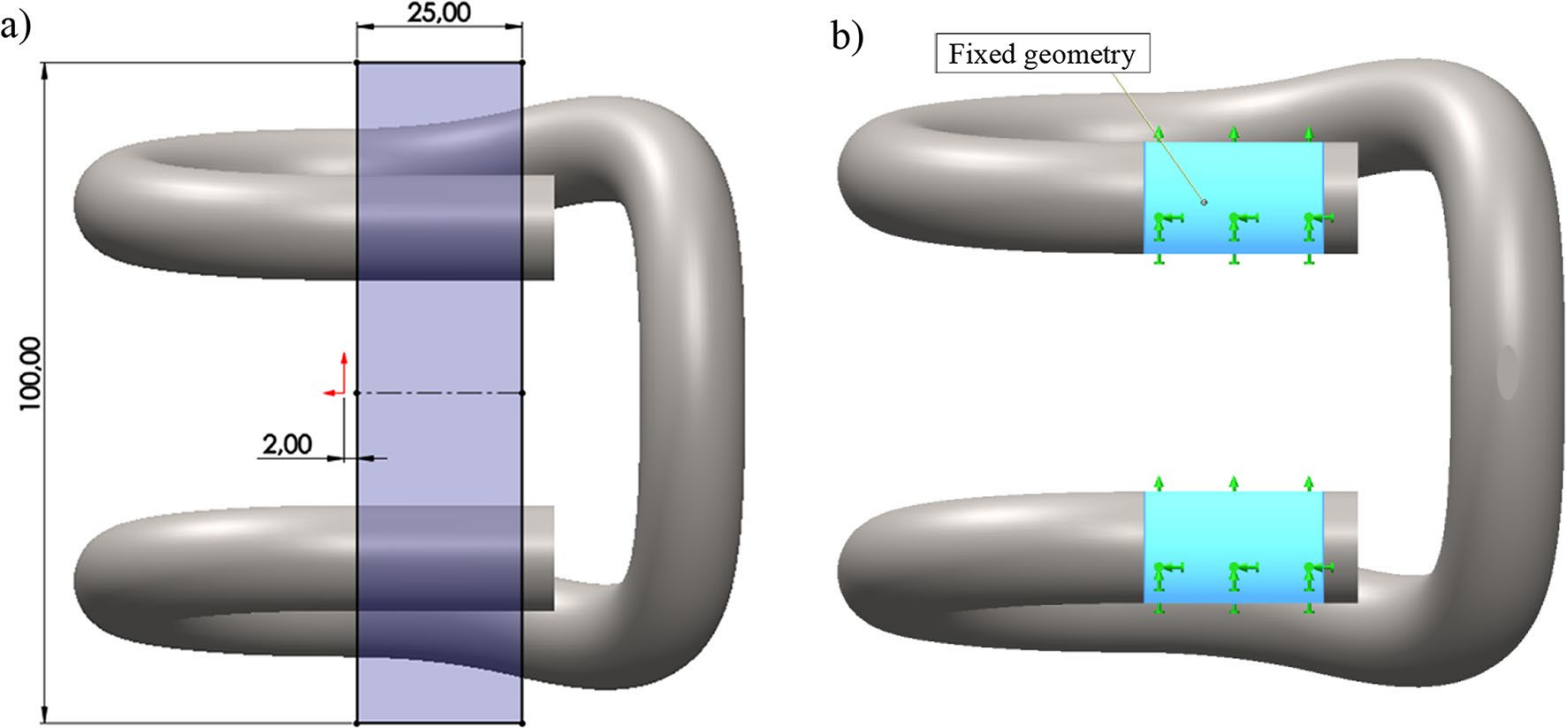
SB 4 tension clamp model used in the FEM analysis: (**a**) area defining the fixture of the clamp, (**b**) immovable area - ‘fixed geometry’.

Numerical calculations were performed in SolidWORKS 2020 for two variants of the clamp geometry. Model A was conformed to the nominal dimensions, and model B reflected the actual dimensions. In both variants, consolidation fixture was applied, on cylindrical surfaces determined by the area shown in Fig. 7a that received all degrees of freedom, for which $\:{U}_{x}\:=\:{U}_{y}=\:{U}_{z}\:=\:{R}_{x}\:=\:{R}_{y}\:=\:{R}_{z}\:=\:0$. In both models, a high-quality curvature-based mesh was applied, providing the best fit of the mesh elements to the elements subject to the 3D scanning. To increase the accuracy of the mesh, integration with 29 Jacobian points was applied. The size of the mesh was adopted at 4 mm. Figure 7b presents the area with the defined clamp fixture.

**Fig. 8:**
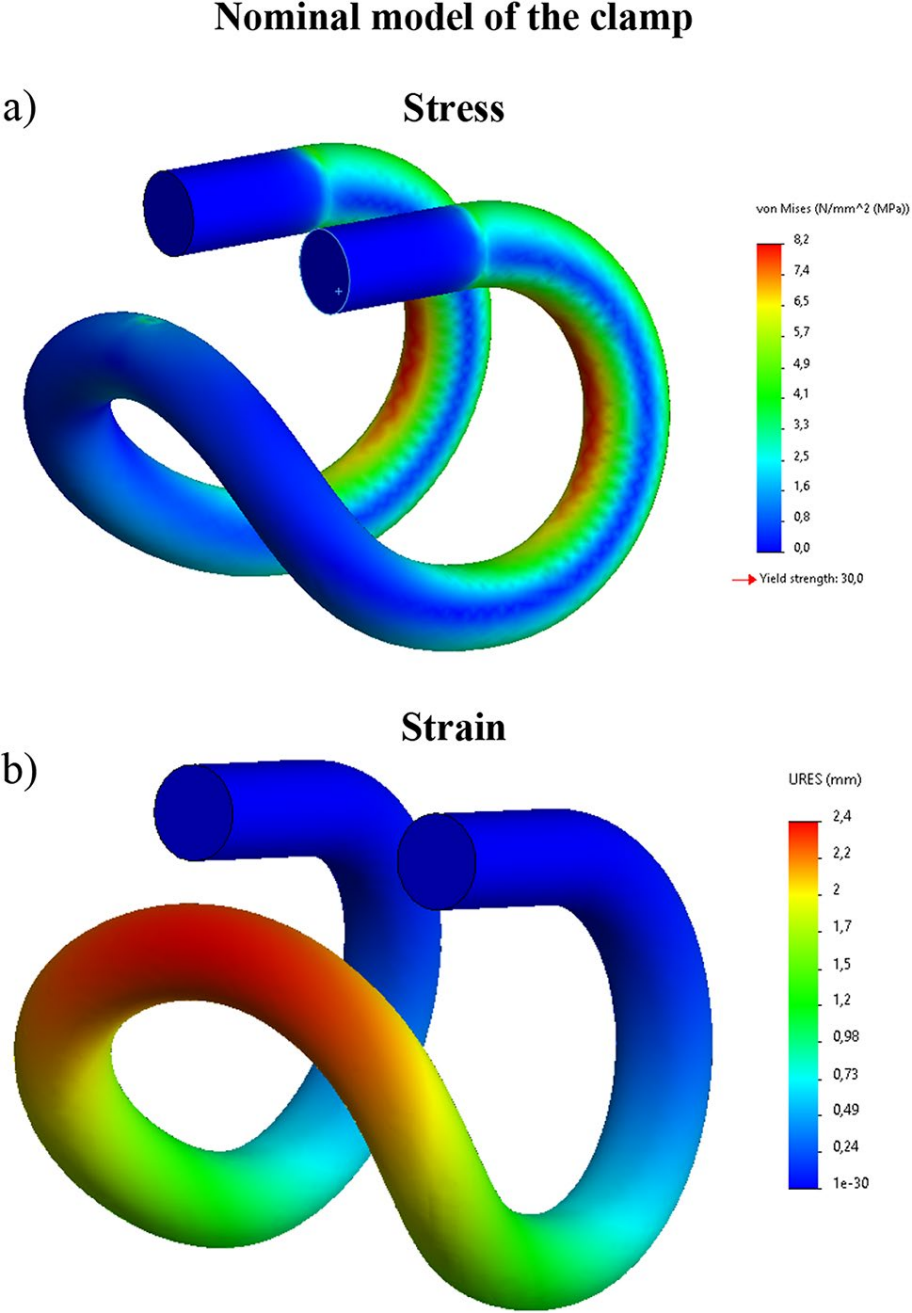
Numerical calculations of a printed tension clamp force 60 [N]; (**a**) stress, (**b**) strain.

**Fig. 9:**
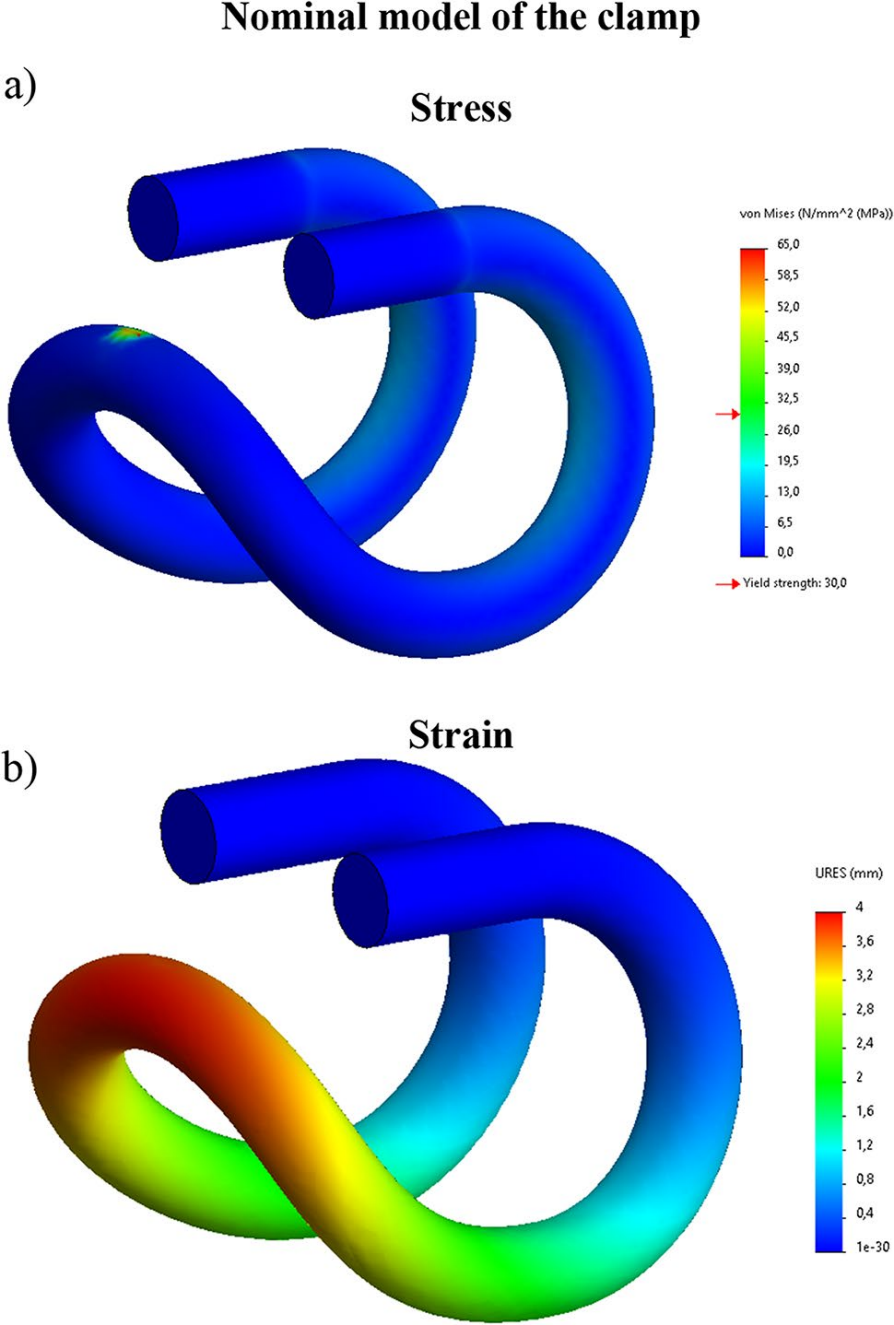
Numerical calculations of a 3D printed tension clamp - forced vertical displacement 4 mm; (**a**) stress, (**b**) strain

Calculations were made for two types of material: a material compliant with 3D A print, for which data from Table 1 were adopted and a material specified in the document ‘Id-109 - steel 50S2’ document^48^. In this publication, the following information is provided: Rm = 1450–1750 MPa, yield strength Re > 1300 MPa. As shown in Fig. 8, the clamp calculations were given in two variants – for the 3D printed clamp: vertical stress − 60 N, defined vertical displacement − 4 mm (Fig. 9).

As shown in Fig. 8, the clamp calculations were given in two variants – for the 3D printed clamp: vertical *force* − 60 N, defined vertical displacement − 4 mm (Fig. 9).

## Results

### Mechanical properties – results

**Fig. 10:**
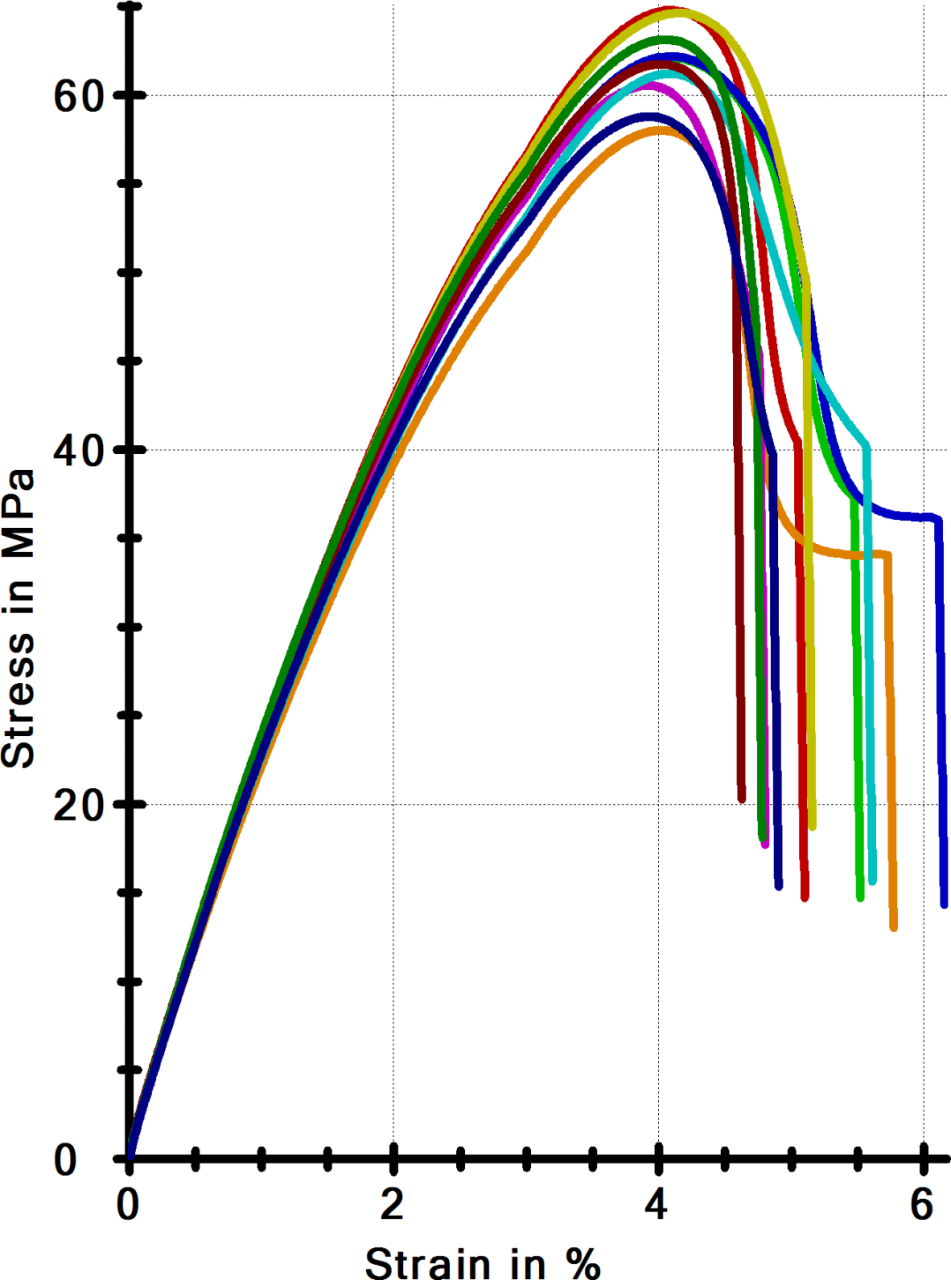
Stress-strain characteristics of the elongation test of the model plastic.

The authors presented the results of their tests carried out on paddle-shaped samples. Figure 10 presents the stress-strain characteristics of the material samples, from which the clamp models were made. Table 2 contains the descriptive statistics of the mechanical parameters obtained in the elongation test according to ISO 527-1:2012. The following quantities were presented: $\:{E}_{t}$ – modulus of elasticity, $\:{\sigma}_{M}$ – tensile strength, $\:{\varepsilon}_{M}$ – strain corresponding to $\:{\sigma}_{B}$, $\:{\varepsilon}_{B}$ – stress at the moment of fracture, $\:{\varepsilon}_{B}$ – stress corresponding to $\:{\sigma}_{B}$, $\:{W}_{M}$ – work of the force on the strain until maximum stress, $\:{W}_{B}$ – work of the force on the strain until fracture. Each of the quantities contained in Table 1 was described with statistical quantities, i.e.: average value - $\:\stackrel{-}{x}$, standard deviation - $\:s$, coefficient of variability - $\:v$.

**Table 2:** Tensile test results.

*N* = 10	*E*_*t*_	*σ*_*M*_	*ε*_*M*_	*σ*_*B*_	*ε*_*B*_	*W*_*M*_	*W*_*B*_
Unit	MPa		%	MPa	%	Nmm	
$\:\stackrel{-}{{x}}$	2630	61.7	4.0	17.6	5.0	2168.10	2908.12
$\:{s}$	87.4	2.03	0.1	1.88	0.4	117.10	250.90
$\nu$	3.33	3.30	2.29	10.65	7.14	5.40	8.63

**Fig. 11:**
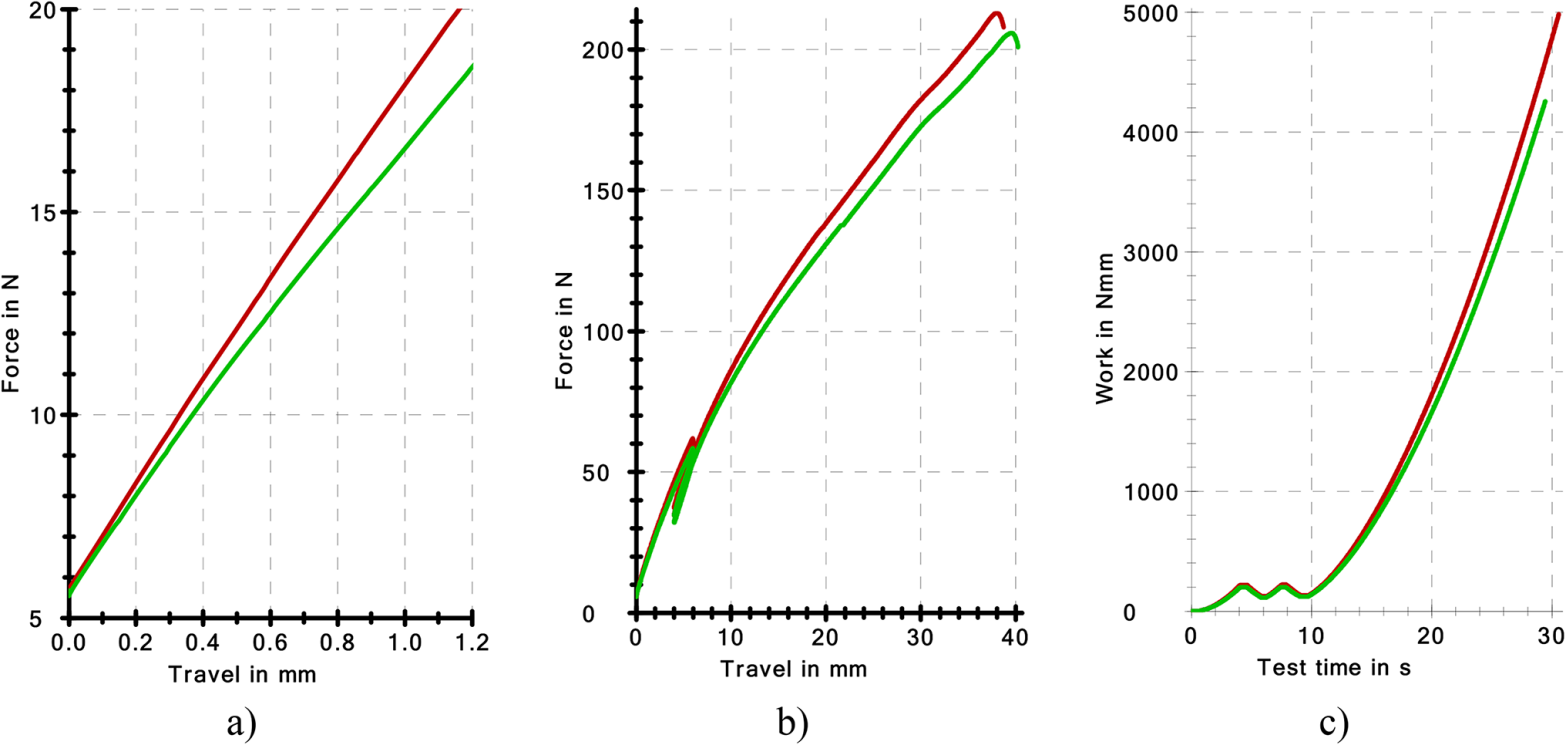
Elastic characteristics of the SB4 tension clamps; (**a**) elastic characteristics (stiffness), (**b**) stress-strain characteristics until fracture, (**c**) work characteristics (energy accumulation) until fracture, nominal SB4 clamp (red– C), SB4 tension clamp, actual (green– INC).

Figure 11a presents the elastic characteristics of the resilient elements compared. The characteristics of the compared clamps were different. Both characteristics of the compared resilient elements have a shape close to linear and degressive to a limited extent. The characteristics of the clamp that are non-conformant with the PKP PLK requirements appear more degressive. Figure 11b presents the course of the work of the clamps until fracture. The courses are similar, yet a more favourable course was observed for the clamps conforming to the nominal shape of PKP PLK. A similar relationship was observed for the work of force on deformation as a function of loading time (Fig. 11c).

Table 3 presents the results that describe the work of the tension clamps under monotonic stress. The table contains the following parameters: N – sample size in the group, $\:{F}_{max}$ – maximum force carried by the clamp, $\:{F}_{Break}$ – force at the moment of clamp fracture, $\:{W}_{{F}_{max}}$ – work of the clamp until maximum stress, $\:{W}_{break}$ - work of the clamp until fracture, $\:{t}_{Test}$ - test time, $\:dL\:\left({F}_{max}\right)$ - bending of the clamp under maximum stress. Each of the quantities in Table 3 was described with the same statistics as in Table 2.

**Table 3:** Results of tension clamp test results carried out under monotonic stress until fracture.

*N* = 3	*F*_*max*_	*F*_*Break*_	*W* *F*_*max*_	*W*_*Break*_	*t*_*Test*_	*dL* (*F*_*max*_)
Unit	N		Nmm		s	mm
Good– C						
$\:\stackrel{-}{{x}}$	203	198	4482.72	4642.12	29.73	36.6
$\:{s}$	17.8	17.8	688.67	692.12	1.46	2.6
$\nu$	8.77	8.98	15.36	14.91	4.9	7.04
Bad – INC						
$\:\stackrel{-}{{x}}$	189	184	3983.43	4081.94	28.55	35
$\:{s}$	17.6	18.5	971.06	1047.23	3.33	5.2
$\nu$	9.3	10.08	24.38	25.66	11.65	14.87

The average strength of the clamps according to the nominal model is slightly higher (by 14 N). A similar dependence was observed for other results: the average work until fracture ($\:{W}_{Break}$) of the samples conforming to the nominal model was higher by approximately 560 Nmm, which gives a difference of the value of this quantity on the level of several percent.

**Fig. 12:**
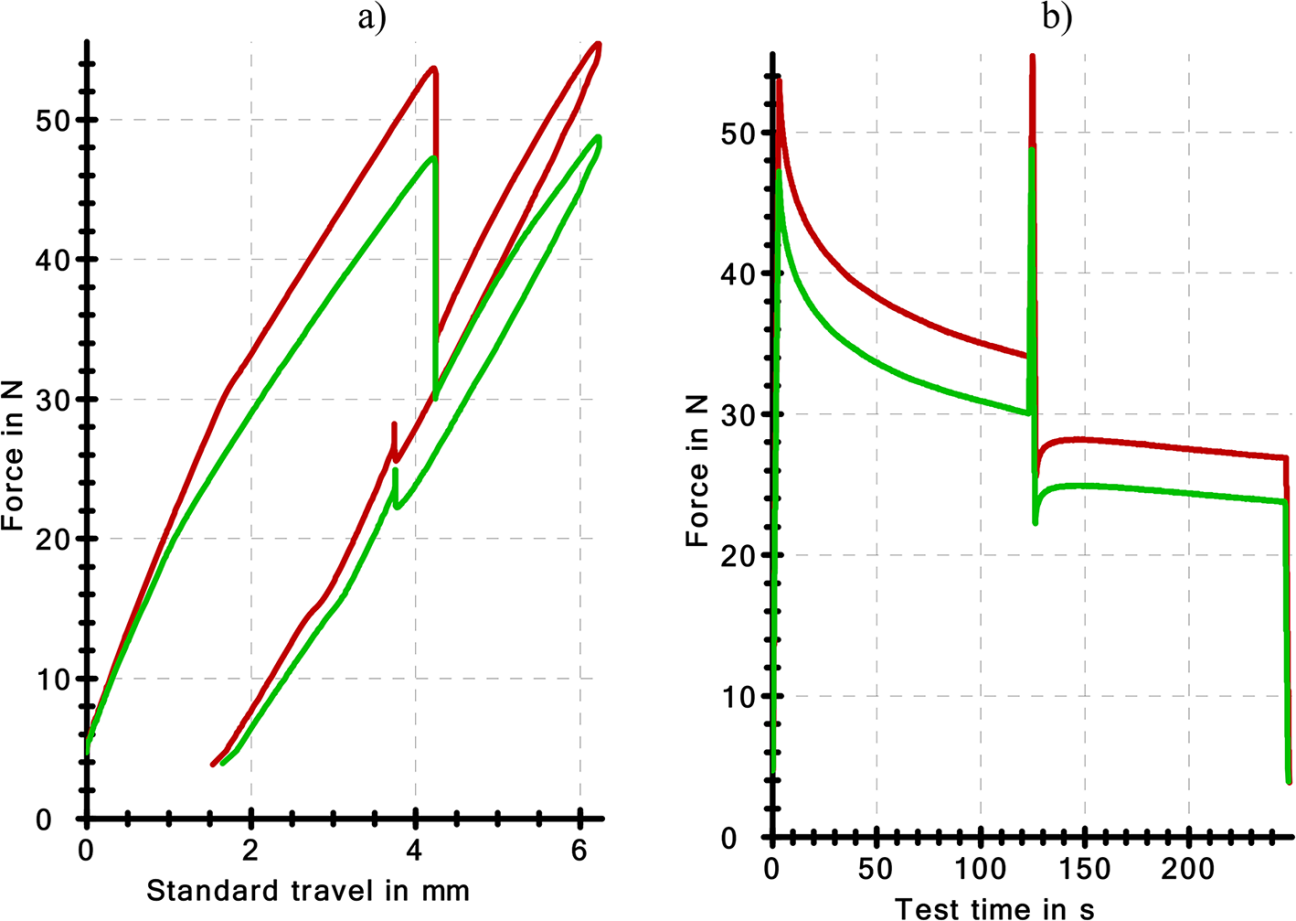
Characteristics of tension clamps under static and dynamic impulse stress; (**a**) SB4 nominal clamp (red- C), (**b**) SB4 actual clamp (green - INC).

Static-dynamic-static tests were carried out according to a scenario: bending 4 –120 s, dynamic impulse bending to 6 mm, release to 4 mm, bending 4 –120 s (Fig. 12). The characteristics obtained indicate an adverse effect of the geometric nonconformity of the tension clamps with the nominal requirements. Figure 12a presents the course of the force as a function of the standard travel of the extensometer arm. Figure 12b presents the characteristics of the force as a function of time. The characteristics of the nominal and the actual clamps do not overlap. The differences are clear. The greatest differences occur in the course of the characteristics in the stress applied to bend the clamp to 4 mm and in the range from 4 mm to 6 mm. The clamps that are conforming to the nominal model are characterised by greater stiffness. A greater force was necessary to obtain the test strains.

**Fig. 13:**
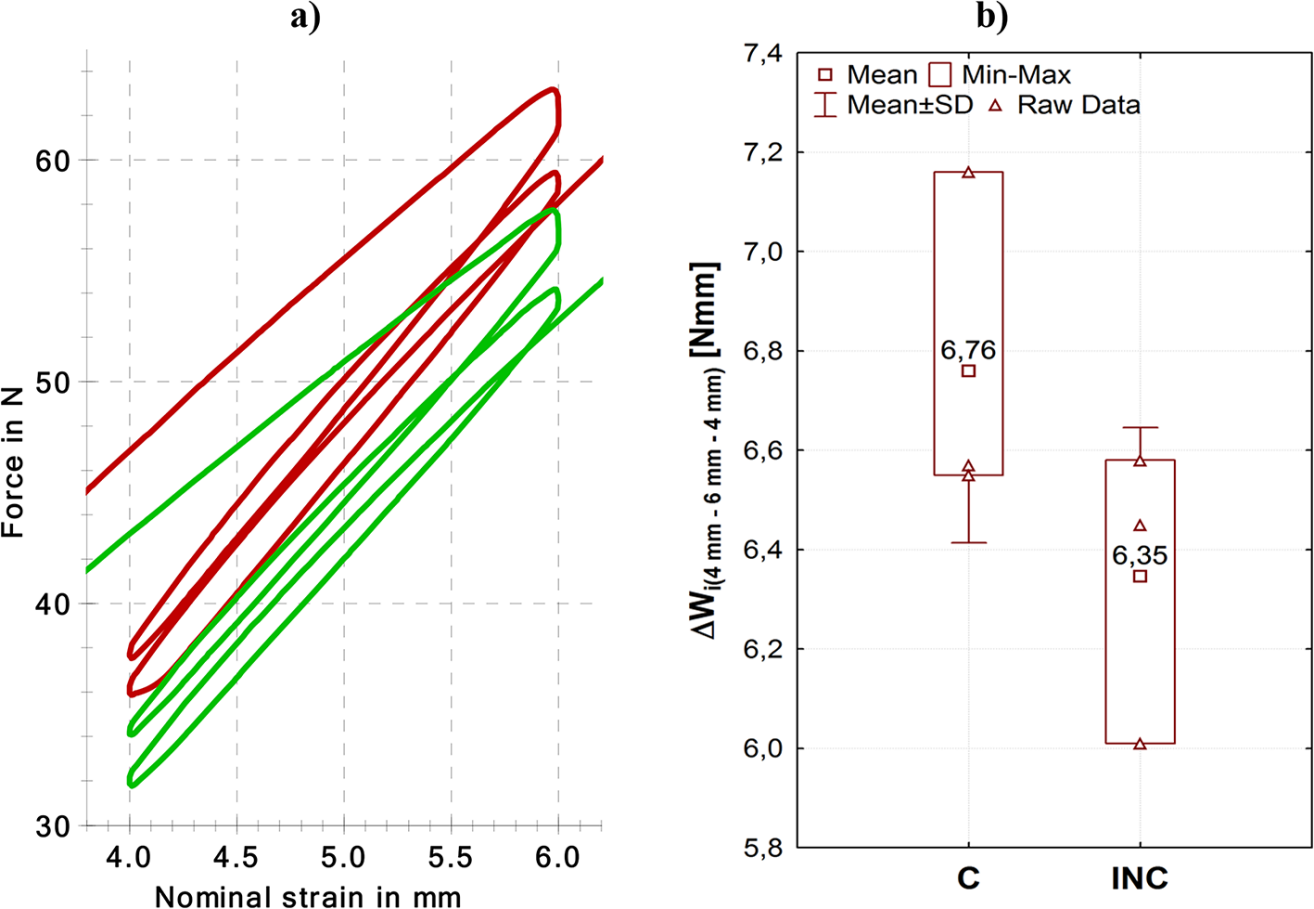
Stress-strain histeresis loop in the range from 4 mm to 6 mm (**a**), frame graph presenting the values of the dissipation energy (**b**).

Table 4 presents the results of the test described with the characteristics presented in Fig. 13. Clamps C are characterised by slightly higher dissipated energy (work) ΔW (approx. 10 Nmm). The value of the dissipated energy is an important parameter that describes the work of the resilient elements^51^. The energy ($\:{W}_{Fapply}$) accumulated by the sample is another significant parameter that describes resilient elements. The value of this quantity is also higher for clamps that conform to the nominal model. It should be noted that the ratio of these two quantities allows for an evaluation of the damping capacity^52^.

**Table 4:** Results of the static-dynamic-static test.

*N* = 3	*F*_*(4mm)Fapply*_	*F*_*(4mm)Frem*_	*F*_*max*_	*W*_*Fapply*_	*W*_*Frem*_	Δ*W*	*dL* (*F*_*max*_)	*dL*(*F*_*min*_ )	*dL* (*F*_*max*_- F_*min*_)
Unit	Nmm			mm					
$\:\stackrel{-}{{x}}$	34.06	26.96	55.1	217.49	116.10	101.39	6.2	1.5	4.7
$\:{s}$	0.26	0.08	0.461	0.92	1.50	2.36	0.01	0.053	0.045
$\nu$	0.77	0.28	0.84	0.42	1.29	2.33	0.13	2.53	0.98
$\:\stackrel{-}{{x}}$	30.19	23.64	49.7	192.03	100.29	91.74	6.2	1.7	4.5
$\:{s}$	0.48	0.30	0.937	4.57	1.54	3.26	0.01	0.1	0.1
$\nu$	1.60	1.27	1.89	2.38	1.53	3.55	0.13	4.19	1.61

In the next stage, the authors analysed the size of the stress-strain hysteresis loop in the 4 –6 mm − 4 mm range. The stress was applied with a velocity of 200 mm/min without interruption, and the stress was of the dynamic impulse nature. In this course of the stress (Fig. 13a) static stress was not taken into account. The result of the test in this respect was the value of the dissipation energy corresponding to the surface area of the hysteresis loop. The larger the hysteresis loop, the greater the ability of the element to dissipate energy and damp vibration. The values of this parameter for the nominal (C) and actual (INC) clamps were similar. However, the average value of the dissipation energy was higher for the nominal clamps (C) (Fig. 13b).

**Fig. 14:**
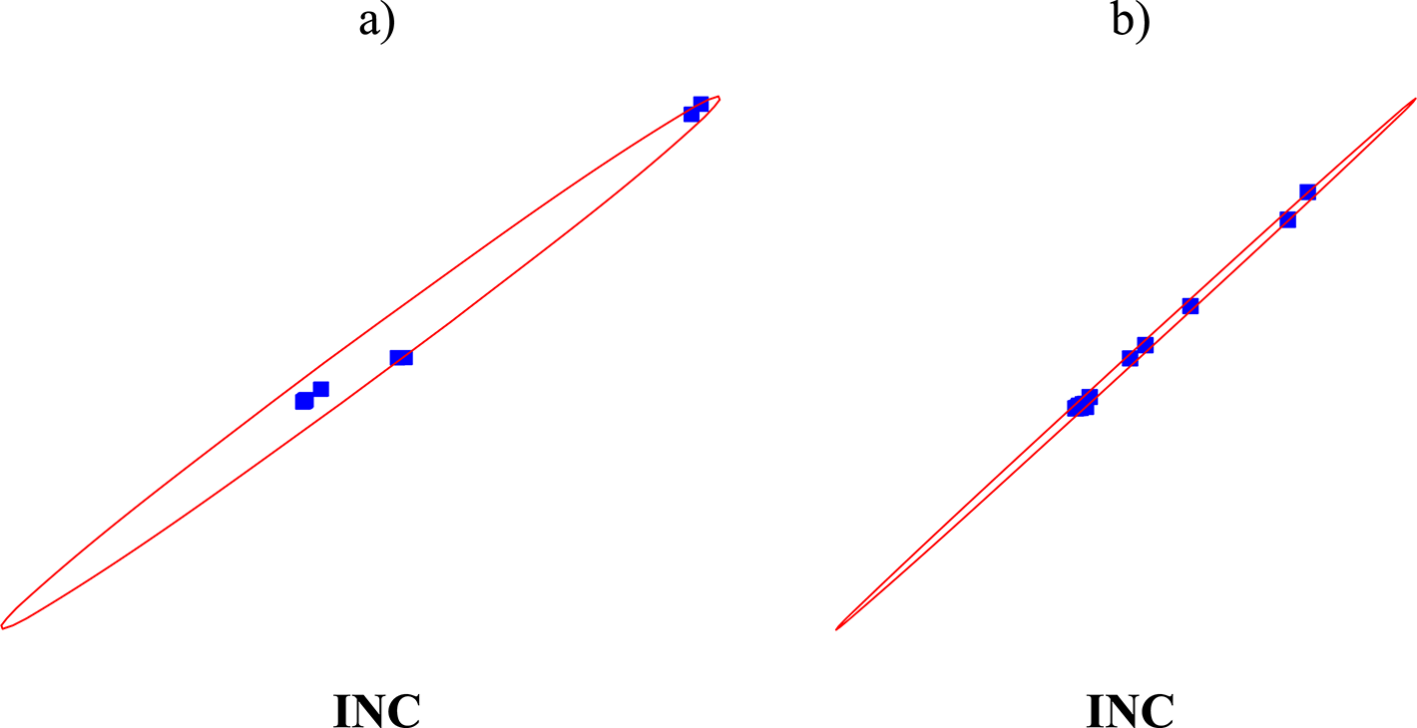
Correlations of the clamp statistical parameters during monotonic (**a**) and static-dynamic-static (**b**) tests.

Assess the importance of the reported discrepancies between the nominal and effective clamp models. An analysis of the mechanical properties of the clamps was conducted using Pearson, Spearman, and Kendall correlation coefficients to evaluate the magnitude and direction of the relationships. These tests confirmed the consistency of the observed differences and ruled out the possibility of random fluctuations. A statistical comparison of the test results for suitable clamps (C) and unsuitable clamps (INC) during both monotonic (a) and static-dynamic-static (b) tests revealed a linear dependence of their parameters, as indicated by the Pearson, Spearman, and Kendall correlation coefficients (Fig. 14; Table 5).

**Table 5:** Value of correlation estimators of the clamp statistical parameters during monotonic.

Correlation coefficient according to	Correlation coefficient values		
	a)	b)	
Pearson	0.995	0.999	
Spearman	0.964	0.943	
Kendall	0.879	0.853	

When the values of the correlation coefficients were examined (Table 5), the following trends were observed. The linear relationship between the statistical parameters was stronger for the (b) test method, as indicated by the Pearson correlation coefficient, which reflects the strength of the linear relationship. In evaluating the monotonicity of the obtained results, the Spearman correlation coefficient demonstrated a higher correlation, capturing only the trends in variable results without accounting for the magnitude of variations. To assess the strength of correlations in the research results, the Kendall correlation coefficient was applied, confirming that the correlations in test (a) were stronger than those in test (b). The research revealed that the nominal and real clamps exhibited comparable, though not identical, hysteresis loops, with variances estimated at approximately 6%. This quantitative methodology enabled the determination of whether the observed disparities were statistically significant or fell within the expected range of variability.

## Discussion

Currently, SB-type fastenings are used to attach rails to prestressed concrete sleepers^53^. This solution complies with European standards (PN-EN 13481 ^54^ and PN-EN 13146 ^55^). The SB fastening system is not just one design solution. During long-term research^53^ it was confirmed that SB4 fastening is characterised by good stress conditions compared to previous SB type fastenings^55^. The mechanical behaviour of the elastic elements of the fastening is translated into the conditions of their operation^56^. During operation, the rail clips are subjected to the combination between toe load (static load) and repeated wheel load (cyclic load), which can cause fatigue failure^57^. Forces generated at the wheel-rail interface are redistributed among other elements of the track system^58,59^. The task of tension clamps is to absorb forces and damp vibrations, as well as to reduce the displacements of the elements of the track system^60^. The mechanical characteristics are also related to a very important feature, which is the ability of the clamp to maintain proper transverse tilt of the rail^61^. Furthermore, the clamp must ensure adequate clamping to the base plate to avoid longitudinal displacements^61^. The investigations presented in this paper confirm that the geometric deviations of the actual clamps (these are to be deemed workmanship defects) translate into the operating characteristics of these elements. Many of the properties mentioned above for SB4 fastenings depend on the operating characteristics of the clamp. Comparative tests have shown that the differences are small, but noticeable. Unfortunately, these differences act against the tension clamp of the actual geometry, different from the nominal model, as has been confirmed in the investigations. Both the physical models and the FEM analysis confirm a higher deformability of the actual clamp models. The results of the static-dynamic-static test show differences to the detriment of the model of actual fastening at a level of approximately 10%. This appears to be small, yet, according to the requirements resulting from^62^, the fastening systems must operate for 3 million cycles and the resistance and stiffness should not drop below 20–25%. In elastic tests, rail clips low toe loads were run-out at 5 × 10^6^ cycles. However, this limit is much lower in the case of impact loads and is limited to even several thousand cycles^57^. If changes in geometry result in the loss of certain properties at a level of 10%, the risk of failure to meet the property loss requirements increases.

Dynamic characteristics are crucial in rail fastening systems^63^. The size of the hysteresis loop obtained in the dynamic tests is different for the investigated models. The average energy of dissipation varies by approximately 6%. This again appears to be a small difference, but it is possible that the change in mechanical properties caused by the geometric imperfection may contribute to the deterioration of the capacity to dissipate the dynamic surplus^64^ generated in motion through the wheel-rail interaction. Furthermore, geometric non-conformance can translate into a different course of fastening fatigue, and, as has been confirmed, fatigue is the main reason for clamp failures^40,42^. The expected immediate strength is significant for the safety factors assumed in structural calculations. However, deformability is also important. The clamp should provide sufficient pressure on the supporting element to limit longitudinal displacements^61^. A different deformability of non-conforming geometry resilient fastenings can result in a different course of rail displacement under operating load. It will be greater than that assumed by designers who assume nominal characteristics of the elements of the rail structure elements in engineering calculations. Higher deformations can also translate into the fatigue process. In the case of resilient fastenings, fatigue is the main cause of failure^37,65^. Research on physical models made on a 2:1 scale, as well as FEM analyses, confirm a higher deformability of the actual clamp models (geometrically non-compliant with the nominal model). The previously mentioned results of the static-dynamic-static test show differences to the disadvantage of the actual model by about 10%. This may not seem much, but according to the requirements arising from legal regulations^66^, fastening systems must operate for 3 million cycles, and resistance and stiffness should not decrease by more than 20–25% during this time. If the change in geometry causes the loss of some properties to a level of 10%, the risk of not meeting the requirements regarding the degree of property loss increases. Accelerated fatigue damage will force railway maintenance services to replace clamps more frequently, which will translate into higher operating costs.

**Fig. 15:**
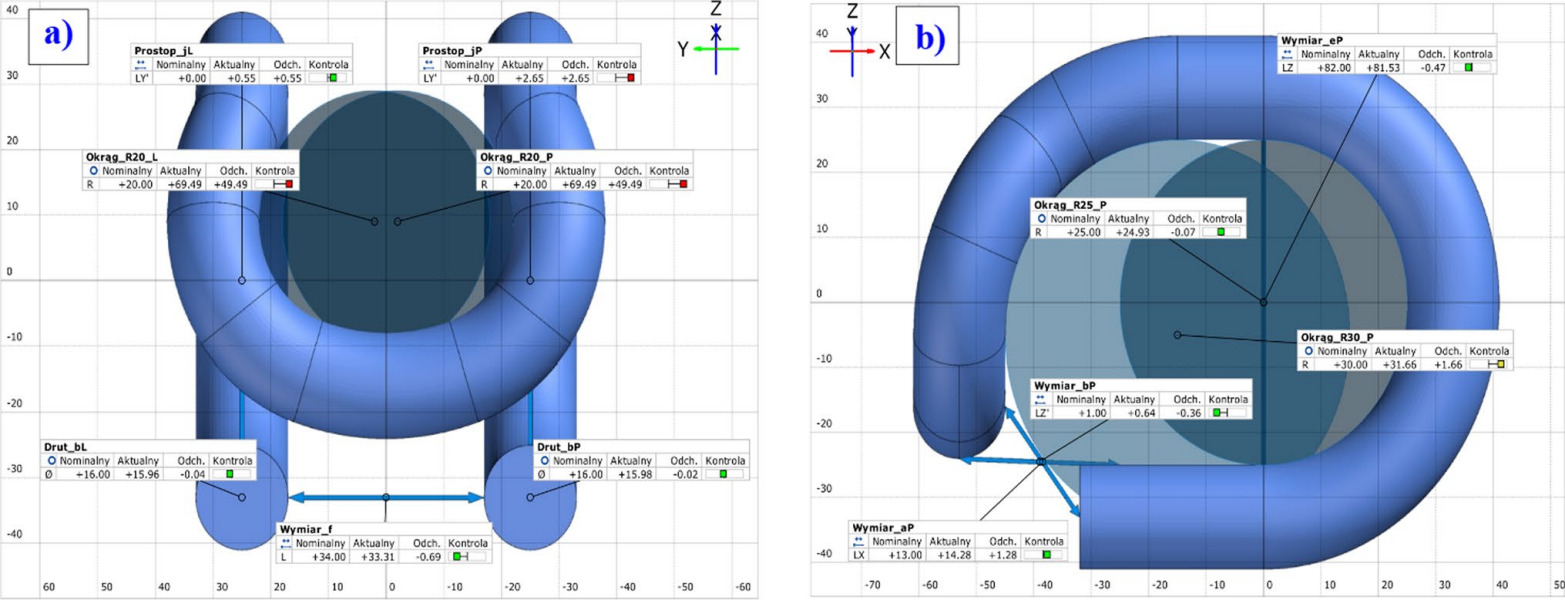
Characteristic dimensions of the SB clamp; (**a**) top view, (**b**) side view

Recently, after completion of the research work, the results of which are published in this article, another work was carried out, the preliminary results of which may constitute the basis for explaining which characteristic dimensions of elastic clamps are of major importance for their operation. The curvature sections of the clip, which are crucial for transmitting operational loads, were assumed to undergo the most significant permanent deformations during installation and operation. These sections were the most stressed and therefore dimensional deviations in these curvature sections of the clips had the most detrimental effect. Figure 15 presents the main dimensions analysed.

Manufacturers are required according to the requirements of PKP PLK^44^ to guarantee expressed in the number of cycles of adjusting and unfastening the clamp on the railway track. The number of cycles guaranteed by the manufacturer should be at least 5 clamp fastenings/unfastenings. Therefore, in the research currently conducted, we analysed permanent assembly deformations of the clamps. For For clamps mounted on the track, deviations in key dimensions were found to increase compared to their dimensions prior to assembly. This suggests that assembly stresses may cause permanent deformations in the clamps. The largest dimensional differences were observed in the main arcs of the clamps, indicating that the highest assembly stresses occur in this part of the spring element. These stresses are likely to exceed the yield strength of the rod material from which the clamps are made. The discrepancies between the actual product geometry and the nominal model likely contribute to this phenomenon.

It is highly probable that changes in geometry affect not only the ability to support the load of the assembly but also the overall operational stresses. Regarding dimension R25, the differences in dimensions after assembly and disassembly were consistently positive for all clamps analysed. However, for dimension R30, three out of five clamps exhibited negative differences. Positive differences were primarily observed in dimensions *a*, *b*, and *e*, suggesting permanent bending of the clamps after assembly on the railway track. Similar positive changes in dimension *b* were reported in the work of Ostromęcka & Aniszewicz^67^. The dimension R25 was found to change further after one year of operation, with positive differences ranging from 0.12 to 0.35 mm. In contrast, dimension R30 exhibited greater variability, with negative differences identified in 13 clamps after one year of operation. This indicates that while this dimension increased for some clamps, it decreased for others. Furthermore, dimensions *a*, *b*, and *e* consistently showed positive differences, which means that these dimensions increased after one year of track operation.

In the analysed spring clamps, the dimensional changes were most prominent in the curved sections of the rod. These findings highlight directions for further research. The approach of assessing changes in spring clamp geometry through 3D scanning, as used in previous work^68^, further validates the utility of this method in operational testing of spring fastenings.

The results described may indicate the need to evaluate the long-term performance and durability of SB4 clamps with geometric non-conformance. However, the research conducted so far has been limited to testing models. In the future, accelerated ageing tests should be considered in the research programme. Various factors should be taken into account, including the operating environment factor^69^. However, such studies would make sense if they were performed on real clamps. Unfortunately, industrially made metal bar clamps in accordance with the requirements of PKP PLK are currently unavailable. This is a limitation of research in this field and should be solved if possible. The solution that aims to overcome these limitations, based on digital twins, is considered appropriate^70^. Furthermore, the synergistic application of FEM analysis for evaluation on scanned models and testing on the elastic clamps machine was presented in Hasap, A., Noraphaiphipaksa, N. & Kanchanomai, C. work^71^. On Polish railways, the examination of track components is carried out by the Diagnostics Centre of PKP.PLK S.A., using specialised rail vehicles. These vehicles are equipped with advanced vision and laser systems that allow for the identification of missing or disengaged clamps. In addition, they conduct comprehensive inspections of the condition of the rails, the subgrade of the railway, and the geometry of the track. These data can serve as the basis for a predictive assessment of clamp damage, using deep learning algorithms^72^, which will be the subject of further consideration by the authors.

Despite the use of elements of the research methodology in a few works, these studies are pioneering and are being conducted by the authors for the first time. There are no comparable studies in the literature that serve as a model suitable for direct transfer to our methodology. Unfortunately, there are no works related to SB fastenings in which a methodology similar to that used in this work would be used. However, the authors bring significant experience from previous research on products made from materials similar in structure and strength^65,67,73–75^. Some team members also have a scientific background in rail transport and railway traction research, including railway tracks (with appropriate literature references). The study also took advantage of the design and operational experience provided by the company that supplied the technical drawings for the rail fastenings. After thorough analysis, the test parameters were carefully selected to reflect the actual working conditions of the fastenings.

The research focused on SB4 tension clamps used to secure railway rails to reinforced concrete sleepers. Initial findings indicate that geometric discrepancies in clamps significantly influence their mechanical properties, particularly elasticity and strength, both critical to the durability and safety of rail infrastructure. Tension clamps enhance rail stability, absorb loads, and mitigate vibrations. The study demonstrated that deviations in clamp geometry directly affect performance and, consequently, the reliability of the entire rail system^45^. Even minor defects in clamps may necessitate more frequent replacements to prevent accelerated wear or further damage, as differences in elasticity and energy absorption between nominal and actual clamps can reach up to 10%. The findings suggest that geometric discrepancies make the elastic properties of the clamps more degressive, meaning that elasticity diminishes more rapidly as the load increases. Addressing these discrepancies during manufacturing could enhance the long-term reliability of railway infrastructure, reducing maintenance requirements and associated costs. This research highlights previously overlooked issues within rail traction systems and underscores the importance of precision in clamp design and manufacturing to improve overall network durability. However, it is essential to note that railway operations adhere to strict industry regulations. Scientific research findings do not directly result in changes to these standards, as implementing such changes requires years of track testing and a strong commitment from the railway sector. These constraints are dictated by the paramount importance of rail traffic safety. Should the opportunity arise, the authors would willingly pursue further operational research on this subject and propose modernized designs for the tested SB clamps.

## Conclusion

Based on the literature review, research, and analyses, several key conclusions were drawn. The dimensions of the clamp arches in the frontal area deviate significantly from the specifications in the technical documentation, altering the shape at the critical anchoring point to the rail. These geometric discrepancies negatively affect the elastic characteristics of the clamps. Although actual clamps exhibit limited variability in work characteristics, their performance is less favourable compared to nominal clamps, particularly in relation to the ∆W parameter, which is crucial for resilient elements. The average strength of the actual clamps is slightly lower than that of the nominal clamps; however, this difference is minimal and may not be statistically significant. The smaller arch radius in the section connecting the arms of the actual tension clamp contributes to less favourable mechanical characteristics, an issue that has been previously highlighted in the context of reducing initial adjustment stress and ensuring adequate fatigue strength.

Deformability differences were also observed between nominal clamps and scaled models of actual clamps, with the most significant variations observed in the curved sections of the rod. These geometric discrepancies are likely the result of manufacturing simplifications that alter the shape of the actual clamps. Although the mechanical impact of these non-conformities is limited, they remain unfavourable and should ideally be avoided. The study used descriptive statistics, controlled experiments, and detailed comparative analyses to ensure statistical robustness. Rigorous sampling, experiment replication, precise measurements, handling of outliers, and application of correlation and comparative tests ensured the reliability of the findings and confirmed the significance of observed disparities between the nominal and actual clamp models.

The integration of statistical techniques with experimental design proved critical to address the challenges inherent in variable production processes. This approach not only provided statistically significant conclusions but also provided a comprehensive understanding of the performance differences between nominal and actual clamp models. These findings form a solid foundation for future research and practical applications that aim to maintain and improving railway infrastructure. They highlight the importance of precision in manufacturing processes to minimise unfavourable variations and ensure long-term reliability of tension clamps.

## Data Availability

The datasets generated during and/or analysed during the current study are available in the Nonconformance-of-the-SB4-tension-clamps repository, https://github.com/cheles74/Nonconformance-of-the-SB4-tension-clamps.

## List of symbols

$\sigma _{M}$: Tensile strength [MPa]
$$F_{M}$$: Maximum force [N]
$\:{A}_{0}$: Initial surface area of the sample [mm^2^]
$\:{\epsilon}_{M}$: Strain corresponding to maximum force [dimensionless]
$\:{\sigma}_{B}$: Stress at the moment of fracture [MPa]
$\:{\epsilon}_{B}$: Strain at the moment of fracture [dimensionless]
$\:{E}_{T}$: Longitudinal modulus of elasticity (Young’s modulus) [MPa]
$\:\text{t}\text{g}{\alpha}_{T}$: Slope of the tangent line to the stress-strain curve [dimensionless]
$\:{W}_{M}$: Tensile work to maximum force [Nmm]
$\:{W}_{B}$: Work until fracture [Nmm]
$\:{\epsilon\:}_{xz}$: Shear strain [dimensionless]
$\:dL\left({F}_{\text{m}\text{a}\text{x}}\right)$: Bending displacement at maximum force [mm]
$\:dL\left({F}_{\text{m}\text{i}\text{n}}\right)$: Bending displacement at minimum force [mm]
$$\:{F}_{\text{apply}}$$: Applied force [N]
$\:{\Delta}W$: Dissipated energy [Nmm]

## Abbreviations

FEM: Finite Element Method
CAD: Computer-Aided Design
PKP PLK S.A. PKP: Polskie Linie Kolejowe S.A.
ZBR: Type of rail fastening
SNR: Signal-to-Noise Ratio
ISO: International Organization for Standardization
PN-EN: Polish Committee for Standardization (in collaboration with European Standards)
OBJET EDEN 500 V: 3D printing system
AHP: Analytic Hierarchy Process
FBG: Fibre Bragg Grating
RGD-720: Photopolymer resin material used in 3D printing
